# Stress during the COVID-19 Pandemic and Emotional Eating Scale Adapted for Children and Adolescents (EES-C) Results in Girls: Polish Adolescents’ COVID-19 Experience (PLACE-19) Study

**DOI:** 10.3390/nu15194197

**Published:** 2023-09-28

**Authors:** Dominika Skolmowska, Dominika Głąbska, Dominika Guzek

**Affiliations:** 1 Department of Dietetics, Institute of Human Nutrition Sciences, Warsaw University of Life Sciences (WULS-SGGW), 159C Nowoursynowska Street, 02-776 Warsaw, Poland; dominika_glabska@sggw.edu.pl; 2 Department of Food Market and Consumer Research, Institute of Human Nutrition Sciences, Warsaw University of Life Sciences (WULS-SGGW), 159C Nowoursynowska Street, 02-776 Warsaw, Poland; dominika_guzek@sggw.edu.pl

**Keywords:** emotional eating, emotional stress, body mass, Adolescent Stress Questionnaire (ASQ), Emotional Eating Scale Adapted for Children and Adolescents (EES-C), adolescents, PLACE-19 Study

## Abstract

Stress related to the outbreak of the COVID-19 pandemic may have caused substantial changes in eating behaviors, and may have been associated with emotional eating, especially in female individuals. The aim of the present study was to analyze the association between stress perceived during the COVID-19 pandemic and emotional eating in girls, within the third phase of the Polish Adolescents’ COVID-19 Experience (PLACE-19) Study. A nationwide sample of 818 Polish female adolescents, aged 15–20, was gathered. The adolescents were recruited to the study based on a random quota sampling procedure. Using a computer-assisted web interview (CAWI) survey, participants filled out the Emotional Eating Scale Adapted for Children and Adolescents (EES-C) to assess the urge to cope with negative emotions by eating, and the Adolescent Stress Questionnaire (ASQ) to assess perceived stress. Additionally, the data concerning body mass, height, as well as body mass change during the COVID-19 pandemic were verified. The groups most susceptible to emotional eating were excessive body mass female adolescents, those who gained weight during the COVID-19 pandemic and those experiencing high stress levels while facing negative emotions. In order to plan effective therapeutic interventions dealing with the issue of emotional eating, both psychological therapy and dietary strategy tailored to the individual should be considered for the indicated susceptible groups.

## 1. Introduction

Emotional states may affect eating behaviors, as they influence the quality and quantity of consumed food products [1]. Appetite especially may be influenced by stress and negative mood, prompting some people to eat more and others to eat less [2]. Such behaviors, known as emotional overeating (or emotional eating, as it is commonly identified with overeating only [3]) and emotional undereating, have been linked with the beginning of body weight problems and eating disorders [4]. On the one hand, negative emotions may trigger a series of physiological reactions which naturally promote a lack of appetite or result in decreased food intake [5]. On the other hand, some people increase food intake, while facing stress, frustration, anxiety or anger, as a coping mechanism [6]. In the study of Macht [6], it was summarized that, on average, 48% of people decrease their food intake, while 30% have increased appetite in response to negative emotions, such as emotional stress. At the same time, other studies indicate that it may be a more serious problem in women than in men [7], and in female adolescents than in male adolescents [8].

A number of studies have focused on emotional eating and resultant energy and macronutrient intake or choice of certain food products [9,10,11]. In the population-based study of Camilleri et al. [9], it was stated that emotional eating was associated with higher intake of high-density snack food products, such as chocolate, pastries, cakes, biscuits and ice cream. A study conducted by Konttinen et al. [10] found that emotional overeating was linked with a consumption of more sweet and non-sweet energy-dense meals, but not with eating fruits or vegetables. A study conducted in an adolescent sample revealed that the emotional eating was associated not only with increased frequency of sweet high-energy-dense food intake, but also salty high-energy-dense foods such as chips [11]. Additionally, emotional eating has been positively associated with waist circumference [12] or body mass index (BMI) [13], so it can be also considered a risk factor for obesity, type 2 diabetes, and hypertension [14].

The COVID-19 pandemic, which began at the end of 2019, was an unprecedented event, which caused not only major changes in daily functioning, including lockdowns and quarantines, but also deterioration in the economic situation of many individuals [15]. Moreover, as it was indicated in the systematic review of Xiong et al. [16], the COVID-19 pandemic was associated with significant psychological distress, including anxiety, depression, and even post-traumatic stress disorder in the general population. In the scoping review by Agrawal et al. [17], it was also highlighted that adolescents were among the most vulnerable groups in terms of mental health problems during the COVID-19 pandemic. Moreover, apart from experienced stress during the COVID-19 pandemic, adolescents are generally exposed to a wide range of stressors in their daily lives, concerning peer relationships, social position, and family conflicts [18]. Moreover, females are in general characterized by a higher level of emotional awareness [19], resulting in their higher emotion recognition ability [20].

As it was indicated in various studies, the COVID-19 pandemic has led to global changes in eating habits [21,22]. In the study of González-Monroy [21], it was stated that during the pandemic the adherence to healthy diets decreased, while simultaneously a higher preference for sweets and ultra-processed food rather than fruits or vegetables was observed. Additionally, some authors have studied the frequency of emotional eating during the COVID-19 pandemic in an adult population and it was noted that a higher frequency of emotional eating was observed in obese individuals [23] and those with higher BMI [24]. In spite of the fact that emotional eating is believed to significantly increase between childhood and adolescence [25], the studies focusing on this population group during the COVID-19 pandemic are scarce.

Therefore, taking into account such a meaningful and stressful period as the COVID-19 pandemic and possible implications in terms of food intake and body mass change, the aim of this study was to analyze the association between stress perceived during the COVID-19 pandemic and emotional eating in girls, within the third phase of the Polish Adolescents’ COVID-19 Experience (PLACE-19) Study.

## 2. Materials and Methods

### 2.1. Ethical Statement

The study was carried out at the Institute of Human Nutrition Sciences, Warsaw University of Life Sciences (WULS-SGGW). The study was conducted according to the guidelines of the Declaration of Helsinki. The study was approved by the Ethics Committee of the Central Clinical Hospital of the Ministry of Interior and Administration in Warsaw (No. 2/2021). Participants and their parents/legal guardians expressed informed consent prior to the study participation.

### 2.2. Population of the Study

The nationwide PLACE-19 Study focused on the life of Polish adolescents (aged 15–20, a standard age for education in the secondary school) during the COVID-19 pandemic, while the whole study was divided into three phases. In the first phase of the study, hygienic and personal protective behaviors were examined [26,27], in the second phase, eating habits were assessed [28,29,30,31,32,33], while in the third phase, the focus was on psychological aspects of eating behaviors [34,35].

The study concerning psychological aspects of eating behaviors was conducted from 21 January to 17 February 2021. The adolescents were recruited to the study using a random quota sampling procedure, with quotas for voivodeships and counties: from each of 16 Polish voivodeships, 5 counties were randomly selected (resulting in 80 counties), and from each of them, 5 secondary schools were randomly selected (resulting in 400 secondary schools). The headmaster of each school received an invitation to take part in the study, and if agreed, a link to the electronic version of the questionnaire form was forwarded to all eligible students.

The following inclusion criteria were established: adolescents attending randomly chosen secondary schools; aged 15–20 years; and informed consent obtained from both students (in case of minor and adult participants) and their parents/legal guardians (in case of minor participants).

The following exclusion criteria were established: any missing or unreliable data in the questionnaire and participation in any previous phase of the PLACE-19 Study.

Finally, a sample within the third phase of the PLACE-19 Study consisted of 1126 adolescents (818 female and 308 male individuals), but within the study of detailed emotional determinants of emotional eating only female participants were assessed, due to the fact that the applied questionnaire, namely the Emotional Eating Scale Adapted for Children and Adolescents (EES-C), includes detailed questions about 26 various emotions, which may have been difficult for boys to complete, as males are in general characterized by a lower level of emotional awareness [19], resulting in their lower emotion recognition ability [20].

### 2.3. Applied Questionnaire

As the present study was conducted during the COVID-19 pandemic, when regular classes at school were suspended and the students had remote learning, data were gathered using the computer-assisted web interview (CAWI) survey, specifically via Google Forms.

The emotional eating was assessed using the EES-C, which was adapted for use in 8–17-year-old children and validated by Tanofsky-Kraff et al. [36], based on the Emotional Eating Scale (EES) developed by Arnow et al. [37]. The EES is a 25-item self-report tool used to determine the urge to cope with negative emotions by eating (overeating). Respondents indicate their desire to eat in response to the specific emotions using five-point scale, with options as follows: ‘no desire’ (rated as 0), ‘small desire’ (rated as 1), ‘moderate desire’ (rated as 2), ‘strong desire’ (rated as 3), and ‘overwhelming urge to eat’ (rated as 4). Higher scores mean higher desire to eat in response to negative moods and states. Additionally, during adapting the form to be used for children and adolescents, the term “happy” was included in the list of emotions in the EES-C, as some studies suggest that eating in response to happiness is associated with binge eating [38].

The EES allows to assess the emotional eating in response to the following emotions: discouraged, guilty, irritated, angry, furious, inadequate, helpless, resentful, frustrated, jealous, rebellious, jittery, on edge, shaky, nervous, excited, uneasy, worried, upset, confused, lonely, bored, sad, blue, and worn out [37]. At the same time, it allows to aggregate the emotional eating within a subgroups for specific factors, as follows: anger (angry, jealous, furious, irritated, resentful, rebellious), anxiety (worried, upset, frustrated, nervous), depression (lonely, inadequate, blue, discouraged, bored, guilty, helpless, confused, sad), somatic (jittery, shaky, on edge, uneasy, excited, worn out), as well as to calculate total value, while the aggregated values are obtained as sum for the included emotions [39].

The adolescent stress was assessed using the Adolescent Stress Questionnaire (ASQ), which was developed and validated by Byrne et al. [40]. The ASQ determines subjective load of stress, including a wide range of adolescent stressors. The questionnaire consists of 56 items which are grouped into 10 stress component scales and include the situations related to: home life, school performance, school attendance, romantic relationships, peer pressure, teacher interaction, future uncertainty, school/leisure conflict, financial pressure, and emerging adult responsibility. Respondents are asked to indicate on a five-point Likert scale, how stressful these situations were for them during the past year, with options as follows: ‘not at all’ (rated as 1), ‘a little stressful’ (rated as 2), ‘moderately stressful’ (rated as 3), ‘quite stressful’ (rated as 4), ‘very stressful’ (rated as 5). Afterwards, a summary score was calculated by adding the individual scores of all 56 items [40] and the participants were classified taking into account their stress level as those declaring the lowest stress level (1st quartile), medium stress level (2nd and 3rd quartile), and the highest stress level (4th quartile).

As neither EES-C, nor ASQ were previously developed for Polish population, they were adopted to be used in Polish according to the recommendations by the WHO [41]. The following stages were applied:

− Forward translation to Polish language (carried out by a Polish native speaker, fluent in English, being a researcher within the studied field of study);
− Backward translation to English language (carried out by a Polish native speaker, fluent in English, being the other researcher than for the previous stage, and having no knowledge about the aim of the study and questionnaire objectives);
− Panel of experts (consisting of Polish native speakers, fluent in English, being the other researchers than for the previous stages, to polish the final version of the questionnaire based on the results obtained in the previous stages of translation, in order to ensure the semantic, idiomatic, cultural, and conceptual equivalent Polish version of the questionnaire).

In addition to the data obtained from the EES-C and ASQ, other questions were also asked in an electronic survey to assess the adolescents’ eligibility for the study and to present the general characteristics of the group, as follows: age, body mass, height, as well as body mass change during the COVID-19 pandemic.

Based on the individuals’ self-declaration of body mass and height, the BMI was calculated, based on the standard calculation [42]. The obtained values were classified as underweight, normal weight, overweight, or obesity, while depending on participants’ age, a different approach was applied. In case of minor participants, the growth reference values were used, while Polish gender-specific and age-specific values were chosen [43], and calculated using Polish OLAF growth charts [44]. The standard BMI growth reference value cutoff points set by WHO were applied, as follows: underweight assigned to BMI < 5th percentile, normal weight assigned to BMI of 5th–85th percentile, overweight assigned to BMI of 85th–95th percentile, and obesity assigned to BMI > 95th percentile [45]. In case of adult participants (at least 18 years), the standard cutoff points for BMI were used as follows: underweight assigned to BMI < 18.5 kg/m^2^, normal weight assigned to BMI of 18.5–24.9 kg/m^2^, overweight assigned to BMI of 25.0–30.0 kg/m^2^, and obesity assigned to BMI > 30.0 kg/m^2^ [42].

The body mass change during the COVID-19 pandemic was determined based on the BMI change between the period of the COVID-19 pandemic (February 2021—period when the study was conducted) and the pre-pandemic period (March 2020 in Poland). For each participant, for the pre-pandemic period the BMI value was calculated, based on the standard calculation [42]. Then, the BMI values or BMI percentile values (depending on whether the participant was an adult or a minor) were compared to state weight loss, weight maintenance, or weight gain during the COVID-19 pandemic.

### 2.4. Statistical Analysis

The sample size was calculated for the population of Polish female adolescents aged 15–20 years, at a 95% confidence level and 5% margin of error while assuming a percentage of 50%. The required sample size was estimated as 384 respondents, so the sample of 818 female adolescents was interpreted as sufficient.

The data distribution was controlled using the Shapiro–Wilk test. The nonparametric Kruskal–Wallis analysis of variance (ANOVA) with post hoc Tukey test was used for comparison of multiple groups.

The statistical analysis was conducted using Statistica version 13.3 (StatSoft Inc., Tulsa, OK, USA), while *p* ≤ 0.05 was indicated as a statistically significant difference.

## 3. Results

The general characteristics of the group of female adolescents studied within the PLACE-19 Study is presented in Table 1. It was observed that the median of age of the studied group was 17.0 (nonparametric distribution). It was stated that most of the female respondents (75.7%) had proper body mass. The highest share of the female respondents declared no body mass change (43.6%) during the COVID-19 pandemic, while similar shares of respondents reported weight loss or weight gain (29.1% and 27.3%, respectively).

The emotional eating assessed on the basis of EES-C stratified by body mass for the group of female adolescents studied within the PLACE-19 Study is presented in Table 2. It was observed that during the COVID-19 pandemic, the emotional eating assessed on the basis of EES-C differed between overweight and underweight participants, as overweight participants more often declared a desire to eat while feeling down (*p* = 0.0007), sad (*p* = 0.0315), helpless (*p* = 0.0382), and upset (*p* = 0.0005) than underweight participants. Moreover, it was also stated that normal weight participants more often declared a desire to eat while feeling they were not doing enough (*p* = 0.0082), than underweight participants and simultaneously, less often declared a desire to eat while feeling worried (*p* = 0.0142) than overweight participants. However, normal weight participants more often declared a desire to eat while feeling happy (*p* = 0.0209) than obese participants.

The aggregated emotional eating factors assessed on the basis of EES-C stratified by body mass for the group of female adolescents studied within the PLACE-19 Study is presented in Table 3. It was observed that during the COVID-19 pandemic, the emotional eating factors assessed on the basis of EES-C differed between overweight and underweight participants, as overweight participants more often declared a desire to eat urged by anxiety (*p* = 0.0038) and general emotions (*p* = 0.0406) than underweight participants. Moreover, it was also stated that normal weight and overweight participants more often declared a desire to eat urged by depression (*p* = 0.0064) than underweight participants.

The emotional eating assessed on the basis of EES-C stratified by body mass change during the COVID-19 pandemic for the group of female adolescents studied within the PLACE-19 Study is presented in Table 4. It was observed that during the COVID-19 pandemic, the emotional eating assessed on the basis of EES-C differed between participants gaining weight and losing weight, as participants who gained weight more often declared a desire to eat while feeling resentful (*p* = 0.0003), discouraged (*p* = 0.0009), shaky (*p* = 0.0059), not doing enough (*p* = 0.0004), excited (*p* = 0.0043), down (*p* < 0.0001), stressed out (*p* < 0.0001), sad (*p* < 0.0001), and uneasy (*p* = 0.0202) than participants losing weight. Moreover, females who gained weight also more often declared a desire to eat while feeling jealous (*p* = 0.0041), worried (*p* < 0.0001), frustrated (*p* = 0.0013), furious (*p* = 0.0001), on edge (*p* = 0.0005), nervous (*p* < 0.0001), angry (*p* = 0.0082), guilty (*p* = 0.0083), bored (*p* = 0.0136), helpless (*p* = 0.0006), and upset (*p* < 0.0001) than participants losing weight.

The aggregated emotional eating factors assessed on the basis of EES-C stratified by body mass change during the COVID-19 pandemic for the group of female adolescents studied within the PLACE-19 Study is presented in Table 5. It was observed that during the COVID-19 pandemic, the emotional eating factors assessed on the basis of EES-C differed between subgroups stratified by body mass change during the pandemic, as participants gaining weight more often declared a desire to eat urged by anxiety (*p* < 0.0001), depression (*p* < 0.0001), somatic (*p* < 0.0001), and general emotions (*p* < 0.0001), than participants maintaining stable body weight and those losing weight. Moreover, it was also stated that participants gaining weight more often declared a desire to eat urged by anger (*p* = 0.0002) than participants maintaining stable body weight and losing weight.

The emotional eating assessed on the basis of EES-C stratified by stress level assessed using ASQ for the group of female adolescents studied within the PLACE-19 Study is presented in Table 6. It was observed that during the COVID-19 pandemic, the emotional eating assessed on the basis of EES-C differed between participants experiencing different stress levels, as participants with the highest stress level more often declared a desire to eat while feeling not doing enough (*p* = 0.0085), jealous (*p* = 0.0298), confused (*p* = 0.0220), angry (*p* = 0.0162), bored (*p* < 0.0001), and helpless (*p* = 0.0060) than participants with the lowest stress level. Moreover, participants with the highest stress level more often declared also a desire to eat while feeling happy (*p* = 0.0006) than participants with the lowest stress level.

The aggregated emotional eating factors assessed on the basis of EES-C stratified by stress level assessed using ASQ for the group of female adolescents studied within the Polish Adolescents’ COVID-19 Experience (PLACE-19) Study is presented in Table 7. It was observed that during the COVID-19 pandemic, the emotional eating factors assessed on the basis of EES-C differed between participants experiencing different stress levels, as participants with the highest stress level more often declared a desire to eat urged by anger (*p* = 0.0049), depression (*p* = 0.0028), and general emotions (*p* = 0.0100) than participants with the lowest and medium stress levels.

## 4. Discussion

Within the conducted study, the groups most susceptible to emotional eating were excessive body mass female adolescents, those who gained weight during the COVID-19 pandemic and those experiencing high stress levels while facing negative emotions. The conducted study is a valuable contribution, as there were no such studies conducted so far during the COVID-19 pandemic. Taking this into account, it presents novel observations, which should be broadened in further studies.

The unexpected outbreak of the COVID-19 pandemic caused severe changes in people’s lifestyles [46]. According to the National Institute of Mental Health and Neurosciences, the sudden lockdowns, resulting in restricted mobility and isolation, led to stress, irritation, boredom, frustration, and even aggressive behaviors [47]. A number of studies indicated that the most common mental health issues experienced during the pandemic were stress, fear, anxiety, anger, and denial, which were observed both in children and adult populations [48,49]. In response to such negative emotions and states, individuals tended to adopt a variety of coping mechanisms in order to mitigate psychological stress [50]. In the period of the COVID-19 pandemic, apart from positive coping strategies, including communicating with friends and family, engaging in hobbies or taking up physical activity, some maladaptive strategies, such as substance use, self-blame, and emotional eating were also observed [50,51,52].

A number of studies indicate that the emotional eating is highly prevalent in overweight or obese female adults [53,54]. In contrast, the frequency of emotional eating in children is very low, as most young children naturally lose their appetite while experiencing stress or negative emotions [55]. Therefore, it is suggested that the emotional eating emerges in the transition period between childhood and adulthood, namely adolescence [56]. In the present study, it was stated that the groups most susceptible to emotional eating were excessive body mass female adolescents, those who gained weight during the COVID-19 pandemic, and those experiencing generally high stress levels while facing negative emotions. Such results are in line with our previous study, as excessive body mass adolescents and those gaining weight during the COVID-19 pandemic were indicated as the most influenced by emotional eating in response to negative emotions during the COVID-19 pandemic [35]. Other studies also confirm that stress is a predictor of emotional eating behavior [57].

It is reported that the responses to stress are variable and there are interindividual physiological and behavioral differences in how an organism perceives stress and in the resulting adaptional or maladaptional processes [58]. Moreover, it is even indicated that different individuals may differently react to the same stressor when it comes to the intensity and duration of the reaction [59], which may also induce various coping mechanisms, including emotional eating [50]. Therefore, this may explain why certain adolescents exposed to high stress gain weight, while others do not. In the study of Wijnant et al. [60], conducted in children and adolescents and concerning stress responsiveness and emotional eating, it was found that after a stress induction, overweight participants and those in chronic stress exhibited increased stress vulnerability and higher intake of sweet or fatty snacks, compared to the proper body mass and low-stress reference group. Therefore, it may be presumed that not all excessive body mass adolescents or those in chronic stress are prone to an acute stressor, and consequently to emotional eating, but only a specific group with both these features, which is additionally characterized by higher stress reactivity and less recovery from stress [60]. Such a finding is consistent with the study of Herhaus et al. [61] carried out in an adult population, in which an acute stressor increased the levels of cortisol only in obese participants, but not in proper body mass ones. Independently from the fact that not all overweight or obese individuals engage in emotional eating, such altered eating behavior is prevalent among excessive body mass individuals [23,53,54].

The findings of the present study highlight the need of addressing the issue of emotional eating in vulnerable groups, namely female adolescents with excessive body mass, those who have a tendency to gain weight in response to stressful events (such as the COVID-19 pandemic) and those who generally have high stress levels and high stress responsiveness. Regaining conscious control of eating is a crucial step to achieve a proper body mass [62]. Behavioral weight loss interventions are often reported not to be effective in overweight or obese individuals [63], as such approaches do not take into account the psychological factors associated with eating, such as emotional eating, which are associated with overweight and obesity [64]. A few psychological interventions, including mindfulness-based interventions or cognitive behavioral therapy, have shown promising results in terms of the reduction of emotional eating and facilitation of weight loss, as they allow individuals to learn acceptance strategies to reduce emotional eating [65]. Moreover, greater awareness of emotions helps individuals improve regulation of eating and make healthier food choices [66]. However, to effectively address the issue of emotional eating, different aspects of therapeutic interventions should be factored in, including psychological approaches and personalized dietary strategies, while planning such interventions [67].

In spite of the fact that the presented study produced novel observations, its limitations should be listed. The study was conducted within the population of female adolescents only, while male adolescents were not studied, and BMI only was assessed, while body composition was not included. Moreover, the study was conducted only in a population from Poland, so the results should be cautiously transferred to other populations. At the same time, as the studied variables (body mass, body mass change during the COVID-19 pandemic, stress level) may be associated, the causality effect cannot be ruled out. Last but not least, the study was conducted using a CAWI method, while the direct methods may provide more reliable observations.

## 5. Conclusions

The groups most susceptible to emotional eating were excessive body mass female adolescents, those who gained weight during the COVID-19 pandemic. and those experiencing high stress levels while facing negative emotions. In order to plan effective therapeutic interventions dealing with the issue of emotional eating, both psychological therapy and dietary strategy tailored to the individual should be considered for the indicated susceptible groups. Moreover, further studies of this area should be conducted in other populations.

## Figures and Tables

**Table 1:** The general characteristics of the group of female adolescents studied within the Polish Adolescents’ COVID-19 Experience (PLACE-19) Study.

Characteristics			Total (*n =* 818)
Age (years)	Mean ± SD		16.7 ± 1.1
	Median (IQR)		17.0 * (2.0)
Body mass	Underweight		29 (3.5%)
	Normal weight		619 (75.7%)
	Overweight		106 (13.0%)
	Obesity		64 (7.8%)
Body mass change	Lost weight		238 (29.1%)
	No body mass change		357 (43.6%)
	Gained weight		223 (27.3%)
Stress level assessed using Adolescent Stress Questionnaire (ASQ)	1st quartile	Mean ± SD	80.99 ± 12.46 82.5 * (20)
		Median (IQR)	
	2nd and 3rd quartile	Mean ± SD	133.85 ± 19.01 134 * (33)
		Median (IQR)	
	4th quartile	Mean ± SD	193.86 ± 19.98 191 * (25.5)
		Median (IQR)	

* Nonparametric distribution (verified using Shapiro–Wilk test; *p* ≤ 0.05).

**Table 2:** The emotional eating assessed on the basis of Emotional Eating Scale Adapted for Children and Adolescents (EES-C) stratified by body mass for the group of female adolescents studied within the Polish Adolescents’ COVID-19 Experience (PLACE-19) Study.

	Underweight (*n* = 29)		Normal Weight (*n* = 619)		Overweight (*n* = 106)		Obesity (*n* = 64)		*p*-Value **
	Mean Score (SD)	Median (IQR)	Mean Score (SD)	Median (IQR)	Mean Score (SD)	Median (IQR)	Mean Score (SD)	Median (IQR)	
Resentful	0.86 ± 0.88	1 (2) *	1.05 ± 1.09	1 (2) *	1.24 ± 1.24	1 (2) *	0.86 ± 0.97	1 (1) *	0.2701
Discouraged	0.59 ± 0.82	0 (1) *	0.8 ± 1	0 (1) *	0.98 ± 1.03	1 (2) *	0.88 ± 1.11	0.5 (1) *	0.1684
Shaky	0.79 ± 0.9	1 (1) *	0.92 ± 0.99	1 (2) *	1.11 ± 1.04	1 (2) *	0.88 ± 1.08	1 (1) *	0.1865
Worn Out	0.69 ± 0.81	1 (1) *	1.1 ± 1.2	1 (2) *	1.06 ± 1.12	1 (2) *	0.78 ± 1.11	0 (2) *	0.0636
Not doing enough	0.86 ± 0.92	1 (2) *^a^	1.55 ± 1.21	1 (1) *^b^	1.53 ± 1.22	1.5 (2) *^ab^	1.27 ± 1.14	1 (2) *^ab^	0.0082
Excited	1.31 ± 1.07	2 (2) *	1.35 ± 1.13	1 (2) *	1.29 ± 1.07	1 (2) *	1.03 ± 0.98	1 (2) *	0.2476
Disobedient	0.79 ± 0.9	1 (1) *	1.08 ± 1.06	1 (2) *	1.01 ± 1.06	1 (2) *	0.88 ± 1.15	0 (2) *	0.1444
Down	0.79 ± 1.21	0 (1) *^a^	1.23 ± 1.34	1 (2) *^a^	1.72 ± 1.41	2 (3) *^b^	1.11 ± 1.39	1 (2) *^a^	0.0007
Stressed out	0.72 ± 1.13	0 (1) *	1.04 ± 1.36	0 (2) *	1.29 ± 1.47	1 (3) *	1.39 ± 1.51	1 (3) *	0.0545
Sad	0.72 ± 1.1	0 (1) *^a^	1.27 ± 1.34	1 (2) *^ab^	1.55 ± 1.44	1 (3) *^b^	1.34 ± 1.47	1 (3) *^ab^	0.0315
Uneasy	0.69 ± 0.85	0 (1) *	0.67 ± 0.9	0 (1) *	0.63 ± 1.01	0 (1) *	0.72 ± 1.16	0 (1) *	0.7031
Irritated	0.9 ± 0.94	1 (1) *	1.06 ± 1.16	1 (2) *	1.05 ± 1.14	1 (2) *	1.11 ± 1.18	1 (2) *	0.9614
Jealous	0.69 ± 0.89	0 (1) *	1.03 ± 1.17	1 (2) *	0.97 ± 1.16	1 (2) *	1.03 ± 1.23	1 (2) *	0.5511
Worried	0.69 ± 1.14	0 (1) *^ab^	0.86 ± 1.1	0 (1) *^a^	1.27 ± 1.35	1 (2) *^b^	1.08 ± 1.33	1 (2) *^ab^	0.0142
Frustrated	0.69 ± 0.93	0 (1) *	0.96 ± 1.13	1 (2) *	1.25 ± 1.3	1 (2) *	1.06 ± 1.27	1 (2) *	0.1163
Lonely	1.07 ± 1.36	0 (2) *	1.46 ± 1.39	1 (3) *	1.7 ± 1.46	1.5 (3) *	1.5 ± 1.54	1 (3) *	0.1465
Furious	0.69 ± 1.04	0 (1) *	1 ± 1.18	1 (2) *	1.04 ± 1.19	1 (2) *	1.19 ± 1.37	1 (2) *	0.3781
On edge	0.93 ± 1.33	0 (1) *	0.89 ± 1.28	0 (2) *	1.29 ± 1.5	1 (2) *	1.06 ± 1.45	0 (2) *	0.0928
Confused	0.79 ± 0.9	0 (2) *	0.91 ± 0.96	1 (1) *	0.99 ± 0.89	1 (2) *	0.78 ± 0.97	1 (1) *	0.2906
Nervous	0.79 ± 1.15	0 (1) *	1.04 ± 1.22	1 (2) *	1.3 ± 1.32	1 (2) *	1.27 ± 1.36	1 (2) *	0.0723
Angry	0.79 ± 1.08	0 (2) *	1 ± 1.19	1 (2) *	1.03 ± 1.25	1 (2) *	1.19 ± 1.32	1 (2) *	0.5818
Guilty	0.41 ± 0.68	0 (1) *	0.75 ± 1.04	0 (1) *	0.84 ± 1.09	0 (1) *	0.77 ± 0.96	0 (1) *	0.2850
Bored	1.38 ± 1.32	1 (2) *	2.02 ± 1.38	2 (2) *	2.03 ± 1.39	2 (2) *	2.19 ± 1.32	2 (2) *	0.0626
Helpless	0.48 ± 0.74	0 (1) *^a^	1.01 ± 1.12	1 (2) *^ab^	1.17 ± 1.21	1 (2) *^b^	1.05 ± 1.28	1 (2) *^ab^	0.0382
Upset	0.66 ± 1.01	0 (1) *^a^	1.05 ± 1.21	1 (2) *^b^	1.51 ± 1.33	1 (2) *^b^	1.27 ± 1.37	1 (2) *^ab^	0.0005
Happy	1.93 ± 1.22	2 (2) *^ab^	1.96 ± 1.19	2 (2) *^a^	1.75 ± 1.16	2 (2) *^ab^	1.5 ± 1.11	2 (1.5) *^b^	0.0209

* Nonparametric distribution (verified using Shapiro–Wilk test; *p* ≤ 0.05); ** compared using Kruskal–Wallis analysis of variance (ANOVA) with post-hoc Tukey test (different letters in rows indicate statistically significant differences).

**Table 3:** The aggregated emotional eating factors assessed on the basis of Emotional Eating Scale Adapted for Children and Adolescents (EES-C) stratified by body mass for the group of female adolescents studied within the Polish Adolescents’ COVID-19 Experience (PLACE-19) Study.

	Underweight (*n* = 29)		Normal Weight (*n* = 619)		Overweight (*n* = 106)		Obesity (*n* = 64)		*p*-Value **
	Mean Score (SD)	Median (IQR)	Mean Score (SD)	Median (IQR)	Mean Score (SD)	Median (IQR)	Mean Score (SD)	Median (IQR)	
Anger	4.72 ± 4.58	4 (8) *	6.22 ± 5.24	6 (7) *	6.33 ± 5.4	6 (8) *	6.25 ± 4.95	6 (7) *	0.4677
Anxiety	2.83 ± 3.25	2 (5) *^a^	3.9 ± 3.77	3 (5) *^a^	5.33 ± 4.41	4 (8) *^b^	4.67 ± 4.4	3 (7) *^ab^	0.0038
Depression	7.1 ± 5.81	6 (8) *^a^	10.99 ± 7.54	10 (11) *^b^	12.5 ± 7.87	12 (10) *^b^	10.88 ± 7.6	10 (11.5) *^ab^	0.0064
Somatic	5.14 ± 4.26	5 (6) *	5.97 ± 4.36	5 (5) *	6.68 ± 4.76	6.5 (6) *	5.86 ± 4.87	4.5 (9) *	0.0626
Total Sum	19.79 ± 16.91	18 (25) *^a^	27.08 ± 19.12	25 (27) *^ab^	30.84 ± 20.42	29 (29) *^b^	27.66 ± 20.01	24.5 (29) *^ab^	0.0406

* Nonparametric distribution (verified using Shapiro–Wilk test; *p* ≤ 0.05); ** compared using Kruskal–Wallis analysis of variance (ANOVA) with post hoc Tukey test (different letters in rows indicate statistically significant differences).

**Table 4:** The emotional eating assessed on the basis of Emotional Eating Scale Adapted for Children and Adolescents (EES-C) stratified by body mass change during the COVID-19 pandemic for the group of female adolescents studied within the Polish Adolescents’ COVID-19 Experience (PLACE-19) Study.

	Lost Weight (*n* = 238)		No Body Mass Change (*n* = 357)		Gained Weight (*n* = 223)		*p*-Value **
	Mean Score (SD)	Median (IQR)	Mean Score (SD)	Median (IQR)	Mean Score (SD)	Median (IQR)	
Resentful	0.86 ± 1.04	1 (1) *^a^	1.06 ± 1.08	1 (2) *^ab^	1.26 ± 1.14	1 (2) *^b^	0.0003
Discouraged	0.63 ± 0.91	0 (1) *^a^	0.85 ± 1	1 (1) *^b^	0.98 ± 1.08	1 (2) *^b^	0.0009
Shaky	0.75 ± 0.87	1 (1) *^a^	0.97 ± 1.02	1 (2) *^ab^	1.07 ± 1.08	1 (2) *^b^	0.0059
Worn Out	0.98 ± 1.14	1 (2) *	1.07 ± 1.15	1 (2) *	1.11 ± 1.23	1 (2) *	0.5124
Not doing enough	1.31 ± 1.15	1 (2) *^a^	1.46 ± 1.14	1 (2) *^a^	1.78 ± 1.31	2 (2) *^b^	0.0004
Excited	1.12 ± 1.07	1 (2) *^a^	1.36 ± 1.03	1 (1) *^b^	1.46 ± 1.24	1 (2) *^b^	0.0043
Disobedient	0.93 ± 1.01	1 (2) *	1.03 ± 1.04	1 (2) *	1.19 ± 1.15	1 (2) *	0.0642
Down	0.94 ± 1.23	0 (2) *^a^	1.26 ± 1.3	1 (2) *^b^	1.62 ± 1.48	1 (3) *^c^	<0.0001
Stressed out	0.82 ± 1.23	0 (1) *^a^	1 ± 1.34	0 (2) *^a^	1.5 ± 1.5	1 (3) *^b^	<0.0001
Sad	1 ± 1.24	0 (2) *^a^	1.27 ± 1.31	1 (2) *^b^	1.65 ± 1.49	1 (3) *^c^	<0.0001
Uneasy	0.52 ± 0.81	0 (1) *^a^	0.72 ± 0.94	0 (1) *^b^	0.74 ± 1.02	0 (1) *^ab^	0.0202
Irritated	0.97 ± 1.06	1 (2) *	1.02 ± 1.11	1 (2) *	1.2 ± 1.28	1 (2) *	0.3048
Jealous	0.84 ± 1.12	0 (1) *^a^	1.02 ± 1.13	1 (2) *^ab^	1.19 ± 1.25	1 (2) *^b^	0.0041
Worried	0.63 ± 0.99	0 (1) *^a^	0.94 ± 1.1	1 (2) *^b^	1.22 ± 1.34	1 (2) *^b^	<0.0001
Frustrated	0.83 ± 1.1	0 (1) *^a^	0.98 ± 1.14	1 (2) *^ab^	1.2 ± 1.23	1 (2) *^b^	0.0013
Lonely	1.17 ± 1.33	1 (2) *^a^	1.45 ± 1.33	1 (2) *^b^	1.86 ± 1.53	2 (3) *^c^	<0.0001
Furious	0.79 ± 1.09	0 (1) *^a^	0.98 ± 1.12	1 (2) *^ab^	1.3 ± 1.35	1 (2) *^b^	0.0001
On edge	0.73 ± 1.18	0 (1) *^a^	0.93 ± 1.27	0 (2) *^ab^	1.26 ± 1.52	1 (2) *^b^	0.0005
Confused	0.81 ± 0.89	1 (1) *	0.89 ± 0.93	1 (2) *	1.03 ± 1.03	1 (2) *	0.0871
Nervous	0.84 ± 1.14	0 (1) *^a^	1.01 ± 1.16	1 (2) *^a^	1.43 ± 1.41	1 (2) *^b^	<0.0001
Angry	0.87 ± 1.14	0 (1) *^a^	0.95 ± 1.14	1 (2) *^ab^	1.24 ± 1.35	1 (2) *^b^	0.0082
Guilty	0.64 ± 0.96	0 (1) *^a^	0.7 ± 0.99	0 (1) *^ab^	0.94 ± 1.14	1 (2) *^b^	0.0083
Bored	1.85 ± 1.38	2 (2) *^a^	1.99 ± 1.34	2 (2) *^ab^	2.22 ± 1.41	2 (2) *^b^	0.0136
Helpless	0.81 ± 1.01	1 (1) *^a^	0.99 ± 1.08	1 (2) *^ab^	1.26 ± 1.29	1 (2) *^b^	0.0006
Upset	0.82 ± 1.13	0 (1) *^a^	1.06 ± 1.15	1 (2) *^b^	1.5 ± 1.4	1 (3) *^b^	<0.0001
Happy	1.78 ± 1.2	2 (2) *	1.98 ± 1.15	2 (2) *	1.88 ± 1.21	2 (2) *	0.0935

* Nonparametric distribution (verified using Shapiro–Wilk test; *p* ≤ 0.05); ** compared using Kruskal–Wallis analysis of variance (ANOVA) with post hoc Tukey test (different letters in rows indicate statistically significant differences).

**Table 5:** The aggregated emotional eating factors assessed on the basis of Emotional Eating Scale Adapted for Children and Adolescents (EES-C) stratified by body mass change during the COVID-19 pandemic for the group of female adolescents studied within the Polish Adolescents’ COVID-19 Experience (PLACE-19) Study.

	Lost Weight (*n* = 238)		No Body Mass Change (*n* = 357)		Gained Weight (*n* = 223)		*p*-Value **
	Mean Score (SD)	Median (IQR)	Mean Score (SD)	Median (IQR)	Mean Score (SD)	Median (IQR)	
Anger	5.26 ± 4.72	4 (7) *^a^	6.06 ± 5.14	6 (7) *^a^	7.37 ± 5.62	6 (9) *^b^	0.0002
Anxiety	3.12 ± 3.47	2 (4) *^a^	3.99 ± 3.71	4 (5) *^b^	5.35 ± 4.39	5 (6) *^c^	<0.0001
Depression	9.16 ± 6.95	8.5 (9) *^a^	10.86 ± 7.28	11 (10) *^b^	13.33 ± 8.12	13 (10) *^c^	<0.0001
Somatic	4.93 ± 3.96	5 (5) *^a^	6.06 ± 4.48	6 (5) *^b^	7.14 ± 4.65	6 (6) *^c^	<0.0001
Total Sum	22.47 ± 17.37	20 (21) *^a^	26.97 ± 18.85	25 (25) *^b^	33.19 ± 20.64	31 (31) *^c^	<0.0001

* Nonparametric distribution (verified using Shapiro–Wilk test; *p* ≤ 0.05); ** compared using Kruskal–Wallis analysis of variance (ANOVA) with post hoc Tukey test (different letters in rows indicate statistically significant differences).

**Table 6:** The emotional eating assessed on the basis of Emotional Eating Scale Adapted for Children and Adolescents (EES-C) stratified by stress level assessed using Adolescent Stress Questionnaire (ASQ) for the group of female adolescents studied within the Polish Adolescents’ COVID-19 Experience (PLACE-19) Study.

	1st Quartile of Stress Level (*n* = 204)		2nd and 3rd Quartile of Stress Level (*n* = 410)		4th Quartile of Stress Level (*n* = 204)		*p*-Value **
	Mean Score (SD)	Median (IQR)	Mean Score (SD)	Median (IQR)	Mean Score (SD)	Median (IQR)	
Resentful	1 ± 1.01	1 (2) *	1.01 ± 1.04	1 (2) *	1.19 ± 1.25	1 (2) *	0.4608
Discouraged	0.87 ± 1.02	1 (2) *	0.78 ± 0.93	1 (1) *	0.87 ± 1.13	0 (1) *	0.7056
Shaky	0.94 ± 0.97	1 (2) *	0.89 ± 0.94	1 (2) *	1.03 ± 1.15	1 (2) *	0.6345
Worn Out	1.08 ± 1.12	1 (2) *	1.01 ± 1.11	1 (2) *	1.13 ± 1.33	1 (2) *	0.7026
Not doing enough	1.34 ± 1.14	1 (2) *^a^	1.46 ± 1.16	1 (1) *^ab^	1.74 ± 1.32	2 (2) *^b^	0.0085
Excited	1.28 ± 1.07	1 (2) *	1.29 ± 1.05	1 (2) *	1.41 ± 1.25	1 (2) *	0.7372
Disobedient	1 ± 0.98	1 (2) *	1 ± 1.07	1 (2) *	1.18 ± 1.13	1 (2) *	0.1526
Down	1.22 ± 1.19	1 (2) *	1.27 ± 1.35	1 (2) *	1.31 ± 1.51	1 (2) *	0.9476
Stressed out	1.06 ± 1.28	1 (2) *	1.09 ± 1.37	0 (2) *	1.11 ± 1.5	0 (2) *	0.7657
Sad	1.14 ± 1.2	1 (2) *	1.34 ± 1.35	1 (2) *	1.34 ± 1.52	1 (3) *	0.3377
Uneasy	0.72 ± 0.93	0 (1) *	0.64 ± 0.88	0 (1) *	0.68 ± 1.03	0 (1) *	0.4203
Irritated	0.89 ± 0.99	1 (1) *	1.07 ± 1.18	1 (2) *	1.2 ± 1.23	1 (2) *	0.0624
Jealous	0.84 ± 0.97	1 (2) *^a^	0.99 ± 1.16	1 (2) *^ab^	1.23 ± 1.31	1 (2) *^b^	0.0298
Worried	0.89 ± 0.98	1 (1) *	0.87 ± 1.14	0 (1) *	1.06 ± 1.36	0 (2) *	0.4003
Frustrated	0.92 ± 1.04	1 (2) *	0.96 ± 1.15	1 (2) *	1.15 ± 1.29	1 (2) *	0.2604
Lonely	1.32 ± 1.19	1 (2) *	1.44 ± 1.37	1 (2) *	1.73 ± 1.65	1 (3) *	0.1059
Furious	0.95 ± 1.07	1 (2) *	0.96 ± 1.17	1 (2) *	1.18 ± 1.33	1 (2) *	0.2439
On edge	0.9 ± 1.14	0 (2) *	0.91 ± 1.3	0 (2) *	1.12 ± 1.55	0 (2) *	0.5013
Confused	0.88 ± 0.95	1 (1.5) *^a^	0.81 ± 0.85	1 (1) *^ab^	1.11 ± 1.11	1 (2) *^b^	0.0220
Nervous	0.96 ± 1.1	1 (2) *	1.05 ± 1.24	1 (2) *	1.26 ± 1.38	1 (2) *	0.1646
Angry	0.93 ± 1.06	1 (2) *^a^	0.92 ± 1.18	0 (2) *^ab^	1.25 ± 1.37	1 (2) *^b^	0.0162
Guilty	0.77 ± 0.94	1 (1) *^a^	0.67 ± 0.99	0 (1) *^ab^	0.89 ± 1.17	0 (2) *^b^	0.0407
Bored	1.6 ± 1.35	1 (3) *^a^	2.03 ± 1.32	2 (2) *^b^	2.39 ± 1.4	3 (3) *^c^	<0.0001
Helpless	0.92 ± 0.97	1 (2) *^a^	0.91 ± 1.06	1 (2) *^ab^	1.31 ± 1.36	1 (2) *^b^	0.0060
Upset	1 ± 1.04	1 (2) *	1.05 ± 1.21	1 (2) *	1.34 ± 1.45	1 (2) *	0.1033
Happy	1.65 ± 1.2	2 (1) *^a^	1.92 ± 1.14	2 (2) *^b^	2.09 ± 1.22	2 (2) *^b^	0.0006

* Nonparametric distribution (verified using Shapiro–Wilk test; *p* ≤ 0.05); ** compared using Kruskal–Wallis analysis of variance (ANOVA) with post hoc Tukey test (different letters in rows indicate statistically significant differences).

**Table 7:** The aggregated emotional eating factors assessed on the basis of Emotional Eating Scale Adapted for Children and Adolescents (EES-C) stratified by stress level assessed using Adolescent Stress Questionnaire (ASQ) for the group of female adolescents studied within the Polish Adolescents’ COVID-19 Experience (PLACE-19) Study.

	1st Quartile of Stress Level (*n* = 204)		2nd and 3rd Quartile of Stress Level (*n* = 410)		4th Quartile of Stress Level (*n* = 204)		*p*-Value **
	Mean Score (SD)	Median (IQR)	Mean Score (SD)	Median (IQR)	Mean Score (SD)	Median (IQR)	
**Anger**	5.61 ± 5.12	6 (7.5) *^a^	5.95 ± 5	5 (7) *^a^	7.23 ± 5.6	6 (8) *^b^	**0.0049**
**Anxiety**	3.76 ± 3.64	4 (5.5) *	3.93 ± 3.76	3 (5) *	4.82 ± 4.43	3.5 (7) *	**0.0750**
**Depression**	10.05 ± 7.59	10 (10.5) *^a^	10.71 ± 7.08	10 (11) *^a^	12.69 ± 8.29	12 (11.5) *^b^	**0.0028**
**Somatic**	5.98 ± 4.78	6 (6) *	5.82 ± 4.01	5 (5) *	6.48 ± 4.92	6 (6) *	**0.4911**
**Total sum**	25.39 ± 20.04	25 (28) *^a^	26.41 ± 17.83	24 (26) *^a^	31.22 ± 21.06	27.5 (29) *^b^	**0.0100**

* Nonparametric distribution (verified using Shapiro–Wilk test; *p* ≤ 0.05); ** compared using Kruskal–Wallis analysis of variance (ANOVA) with post hoc Tukey test (different letters in rows indicate statistically significant differences).

## Data Availability

Data provided on request.
